# SnoN upregulation ameliorates renal fibrosis in diabetic nephropathy

**DOI:** 10.1371/journal.pone.0174471

**Published:** 2017-03-28

**Authors:** Lirong Liu, Mingjun Shi, Yuanyuan Wang, Changzhi Zhang, Bo Su, Ying Xiao, Bing Guo

**Affiliations:** 1 Department of Clinical Hematology, Affiliated Hospital of Guizhou Medical University, Guiyang, Guizhou, China; 2 Department of Pathophysiology, Guizhou Medical University, Guiyang, Guizhou, China; 3 Department of Respiratory Medicine, People’s Hospital of Guizhou Province, Guiyang, Guizhou, China; 4 Department of Pathology, Nanyang Central Hospital, Nanyang, Henan, China; Faculty of Medicine & Health Science, UNITED ARAB EMIRATES

## Abstract

Progressive reduction of SnoN is associated with gradual elevation of TGF-β1 during diabetic nephropathy progression, suggesting SnoN to be a possible mediator of TGF-β1 signaling, with potential therapeutic benefits against TGF- β1 –induced renal fibrosis. To characterize SnoN for its role in renal fibrosis, we assessed SnoN expression patterns in response to high glucose stress, and evaluated the effects of upregulating SnoN on renal fibrosis. High glucose stress induced significantly elevated SnoN, TGF-β1, and Arkadia transcription; however, significantly reduced SnoN protein levels were observed under these conditions. Upregulating the SnoN protein was achieved by Arkadia knockdown, which resulted in inhibited high glucose-induced epithelial-mesenchymal transition (EMT) in renal tubular cells, the onset phase of renal fibrosis. Alternatively, EMT was suppressed by dominantly expressed exogenous SnoN without interfering with TGF-β1. Overall, renal SnoN upregulation ameliorates renal fibrosis by relieving high glucose-induced EMT; these findings support a translational approach targeting SnoN for the treatment of diabetic nephropathy.

## Introduction

Diabetic nephropathy (DN) is a severe microvascular complication of diabetes, and evolves progressively toward end-stage renal failure (ESRF). With increasing incidence, DN is the major contributor to chronic renal failure and leading cause of death in diabetic patients [1]. Renal tubulointerstitial fibrosis is one of the distinct features for pathological diagnosis of DN. Multiple factors, including vasoactive substances, chemokines, cytokines, and growth factors, are involved in hyperglycemia triggered DN progression, with TGF-β1 and its co-factors playing critical roles [2–4].

The significant increase of TGF-β1 in various renal cells, such as mesangial-, tubular epithelial-, vascular endothelial-, smooth muscle-, and interstitial cells, is associated with pathological diagnostic characteristics [5–6]. Signal transduction from TGF-β1 through its kinase Smad promotes renal epithelial-mesenchymal transition (EMT), and consequently causes extensive renal fibrosis [7–9]. The profibrotic role of TGF-β1 suggests that blocking of the TGF-β1/Smad signaling pathway may represent an attentive approach in preventing DN progression.

Previous reports demonstrated that SnoN interacts with Smad to block TGF-β1/Smad signaling, suppressing the transcriptional activation of TGF-β1 target genes; therefore, SnoN negatively regulates the physiological effects of TGF-β1 [10–12]. SnoN is also affected by TGF-β1/Smad signaling pathway effectors, either positively or negatively. Thus, the approach of upregulating SnoN appears valuable and worthy of exploration. In this study, we confirmed the significant negative correlation between SnoN and TGF-β1, designed two strategies of SnoN upregulation (knocking down a negative regulator of SnoN or introducing exogenous copies of SnoN), and demonstrated the suppression of renal tubular cell EMT due to SnoN upregulation, which would consequently result in protection from renal fibrosis.

## Materials and methods

### Ethics statement

This study was approved by the Animal Care Welfare Committee of Guizhou Medical University (authorization number 1301027). All animal studies were performed in compliance with the regulations set forth by the Administration of Affairs Concerning Experimental Animals.

### Diabetic rat model and grouping

Male Sprague-Dawley (SD) rats, clean grade (180± 20 g), were provided by HFK Bioscience, Beijing. The diabetic(DM) rat model was established by intraperitoneal injection of STZ at 55 mg/kg. Blood glucose levels were monitored 48h post injection. Rats with fasting blood glucose levels ≥16.7 mmol/L were considered to be diabetic, and assigned to the DM group. Rats in the normal control(NC) group received sterile citric acid/sodium citrate buffer only. Each group had 10 rats. The animals were euthanized at 16 weeks, with samples collected for subsequent analysis.

### Cell culture and grouping

Rat (NRK52E) and human (HK-2) renal tubular cells were kindly provided by Professor Limin Lu, Fudan University, China, who purchased them from the Cell Bank of Chinese Academy of Sciences (Shanghai, China). NRK52E cells were maintained in a 37°C, 5% CO2 incubator, in DMEM containing 10% FBS, 100 U/ml penicillin, and 100μg/ml streptomycin. HK-2 cells were cultured in F12/DMEM containing 10% FBS, 1.5 mM L-Glutamine, 100 U/ml penicillin, and 100μg/ml streptomycin. Cells were initially seeded at 2x10^5^ per flask. At 80% confluency, the culture conditions were switched to serum-free for 24h to synchronize cell growth. The cells were then grouped as follows. For NRK52E cells, the NG (normal glucose level) group was incubated with 2% FBS+DMEM+5.5 mmol/L D-Glucose, while HG (high glucose level) group cells received 2% FBS+DMEM +25 mmol/L D-Glucose. Sampling was performed at 2h, 12h, 24h, 48h, and 72h, respectively. For HK-2 cells, the NG and HG groups were sub-divided in siRNA-574 transfection, siRNA-NC oligo transfection, and Blank control groups.

### Histochemical, immunohistochemical, immunofluorescent staining and western blot

Masson trichrome staining was performed accorded to the protocol provided by the manufacturer (Sigma-Aldrich). To measure renal fibrosis quantitatively, the collagen tissue area was evaluated using Image ProPlus Software (Media-Cybernetics, Silver Spring, MD) by drawing a line around the perimeter of the positive staining area, and the average ratio to each microscopic field (400×) was calculated and graphed.

Tissue specimens were fixed immediately in 4% buffered paraformaldehyde for paraffin embedding. Immunohistochemistry and immunofluorescence were performed as described previously [13]. Cells were seeded into a 6-well plate on coverslips and treated as described above. After fixation with 4% paraformaldehyde, the cells were blocked with goat serum, and incubated overnight at 4°C with E-cadherin and α-SMA primary antibodies. This was followed by incubation with FITC/TRITC-labeled secondary antibodies. Finally, the cells were counterstained with DAPI and observed under a microscope (Olympus FV1000, Olympus, Japan).

For Western blot, total protein extracts were obtained after lysing the cells in RIPA buffer. Equal amounts of total protein were separated by SDS-PAGE, and protein bands were electro-transferred onto a PVDF membrane (Millipore, Billerica, USA). After blocking in TBS-T containing 5% non-fat dry milk, primary antibodies were added for overnight incubation at 4°C. Blots were then incubated with horseradish peroxidase-conjugated secondary antibodies at room temperature for 1h. Band intensities were quantified on a Bio-Rad gel imaging system (BIO-RAD, Hercules, USA) with the Quantity one 4.6 software (Bio-Rad, USA). β-actin was used as an internal control. Anti-SnoN, anti-Arkadia, anti-TGF-β1, and anti-E-cadherin primary antibodies were purchased from Santa Cruz (USA); anti-α-SMA primary antibody was from Bioss (Beijing, China); anti-fibronectin (FN) and anti-β-actin primary antibodies, as well as FITC/TRITC-labeled secondary antibodies and DAPI were from Boster (Wuhan, China).

### siRNA synthesis and transfection

siRNAs targeted to Arkadia, including siRNA-574, -879, -1347, and -1744, as well as siRNA-NC-FAM and scramble control siRNA-NC-oligo were synthesized by Corning. The parameters for optimizing RNA interference and individual siRNA scores were preliminarily determined (data not shown), and siRNA-574 had the highest score for silencing the Arkadia gene. Transfection was performed using Lipofectamine 2000 (Life Tech) following the manufacturer’s instructions.

### Lentivirus preparation and transduction

**Fig 1:**
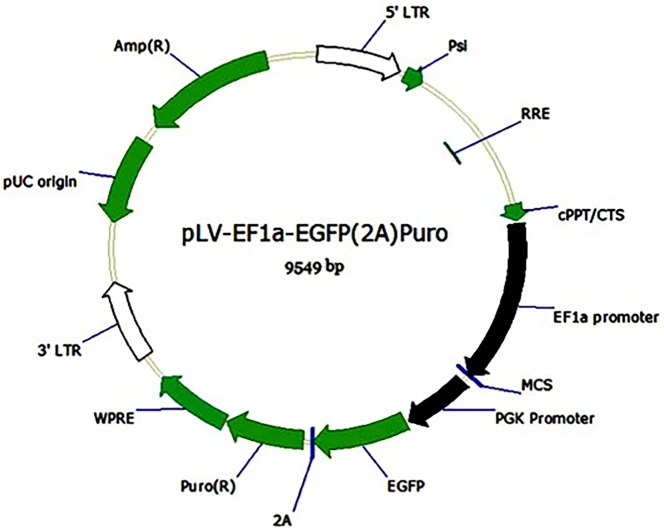
Map of the pLVEF1a-EGFP(2A)-Puro vector for lentivirus delivery.

The SnoN coding sequence (CDS) was retrieved from GenBank (NM_005414.4), synthesized, and inserted into the pLV-EF1a-EGFP(2A)-Puro vector (Fig 1). Lentiviral packaging was carried out by GenePharma (Shanghai, China). The transduced HK-2 cells were selected in media containing puromycin at 0.6μg/ml for 7 days, yielding a stable cell line named HK-2-V1991. An empty vector without SnoN insert was used as control to generate a stable puromycin-resistant cell line named HK-2-NC.

### Enzyme-linked immunosorbent assay for FN detection

Enzyme-linked immunosorbent assay (ELISA) was performed with a customized kit from Eiaab (Wuhan, China), following the manufacturer’s instructions.

### RNA extraction and quantitative real-time PCR analysis [13]

Total RNA was extracted using TRIzol Reagent (Invitrogen, Carlsbad, USA) according to the manufacturer’s protocol. First strand cDNA was synthesized with Takara RNA PCR kit (Baoshengwu, Dalian, China) according to the manufacturer’s instructions. Gene expression levels were measured by real-time RT-PCR using SYBR Select Master Mix (BIO-RAD, Hercules, USA), on a CFX 96^TM^ Connect Real-Time system (BIO-RAD, Hercules, USA). Primers were designed with the DNAMAN software and synthesized by Generay Biotech Co., Ltd. (Shanghai, China). β-actin was used for normalization. Primer sequences are listed in Table 1.

**Table 1:** Primer sequences.

Gene	Primer sequence		Product
TGF-β1	Forward	5′- GAAAGCCCTGTATTCCGTCTCC-3′	129bp
	Reverse	5′- GCAACAATTCCTGGCGTTACCT -3′	
SnoN	Forward	5′- TGTCTGAGAAACATGGTCACCTTCC -3′	92bp
	Reverse	5′- AGGGAGCGTCGGGCTGAACATA-3′	
Arkadia	Forward	5′- CCTCACATCCGTTACATTTCTT-3′	140bp
	Reverse	5′- AGAGTGTATGTGGCAATGCGTT -3′	
α-SMA	Forward	5′- TGAGAAGAGTTACGAGTTGCCTG -3′	140bp
	Reverse	5′- TGATGCTGTTGTAGGTGGTTTCAT -3′	
E-cadherin	Forward	5′- ACAAAGACAAAGAAGGCAAGGTTT -3′	148bp
	Reverse	5′- AGAGTGTATGTGGCAATGCGTT -3′	
FN	Forward	5′- CCATTGCAAATCGCTGCCAT -3′	153bp
	Reverse	5′- AACATTTCTCAGCTATTGGCTT -3′	
β-actin	Forward	5′- ACCACCATGTACCCAGGCAT -3′	169bp
	Reverse	5′- CCGGACTCATCGTACTCCTG -3′	

### Statistical analysis

Each experiment was performed at least three times. Group comparisons were performed by one-way analysis of variance (ANOVA), followed by the Bonferroni multiple comparison test (more than two groups) or Student's t-test (two groups). Correlation coefficients were determined by linear correlation analysis. P<0.05 was considered statistically significant.

## Results

### High glucose stress promotes the EMT in rat renal tubular cells and fibrogenesis in rat diabetic model

**Fig 2:**
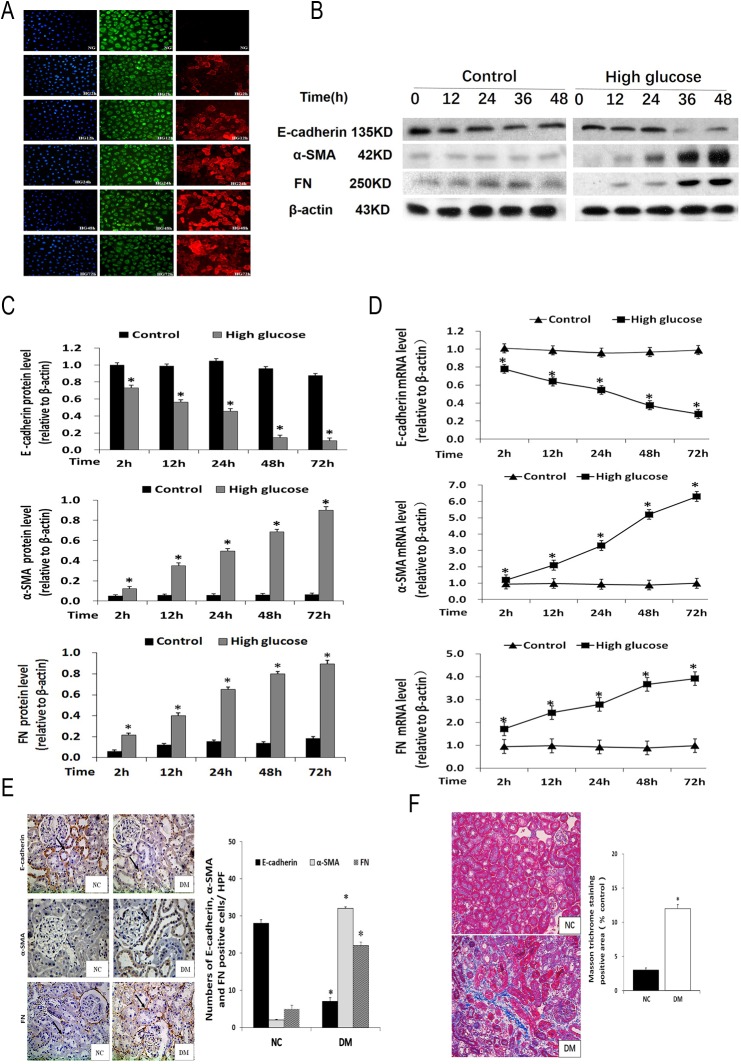
High glucose stress promotes the expression of EMT related genes in renal tubular cells and a rat diabetic model. **A,** E-cadherin (green) and α-SMA (read) levels in NRK52E cells assessed under an inverted fluorescence microscope (blue: nucleus) (200×). **B, C,** E-cadherin, α- SMA and FN protein amounts determined by Western blot in NRK52E cells treated with 25 mmol/L glucose for 2, 12, 24, 48 or 72h. *P<0.05 vs respective NRK52E cells treated with 5.5 mmol/L glucose for the same time. **D,** E-cadherin, α-SMA and FN mRNA levels determined by qRT-PCR in NRK52E cells treated with 25 mmol/L glucose for 2, 12, 24, 48 or 72h. *P<0.05, vs. respective NRK52E cells treated with 5.5 mmol/L glucose at 2h. **E,** E-cadherin, α-SMA and FN protein expression levels in kidney tissues assessed by immunohistochemical staining (400×). Data are represented as the means±SDs (n = 6).*P<0.05, vs. respective normal control group. **F,** Photomicrographs illustrating Masson trichrome staining of kidney tissue (400×) and the Masson trichrome–positive tubule interstitial area (blue) relative to the whole area from ten random cortical fields was analyzed. Data are represented as the means±SDs (n = 6). *P<0.05, vs. respective normal control group. *Notes*: NC, control group; NG, normal glucose group; HG2-72h, high glucose (2h, 12h, 24h, 48h, and 72h) group; DM, diabetes group.

The expression patterns of E-cadherin, α-SMA, and fibronectin (FN) exhibit distinct features during EMT in DN progression [14–16], which were analyzed in this study. The renal tubular NRK52E cells, cultured in high glucose medium, showed decreased E-cadherin levels and increased α-SMA and FN amounts, in a time dependent manner. In contrast, the cells cultured at a normal glucose level showed abundant E-cadherin but sporadic α-SMA, with no significant changes in gene expression patterns over time. This was demonstrated through multiple approaches, including immunofluorescence, Western blot, and qRT-PCR, which gave consistent results (Fig 2A–2D).

We analyzed EMT-related factors in a rat diabetic model. As demonstrated by immunochemistry, in the DM group, E-cadherin levels in renal tubular epithelial cells (RTECs) were reduced significantly, while α-SMA amounts were markedly increased; in addition, intercellular FN levels were increased significantly. In the normal control group (NC group), however, high levels of E-cadherin were detected in RTECs, with negligible amounts of α-SMA and FN detected (Fig 2E).

Furthermore, collagen fibrils are extensively deposited within the interstitial space thaas a consequence of myofibroblast activation in the DM group at week 16. That was shown by an increase in positive areas of Masson trichrome staining. Semi-quantitative analysis of Masson trichrome–positive areas revealed about a three-fold increase in deposition of ECM components in the DM group compared with NC group (Fig 2F).

### High glucose stress suppresses SnoN while activating TGF-β1 and Arkadia in renal tubular epithelial cells and diabetic rats

**Fig 3:**
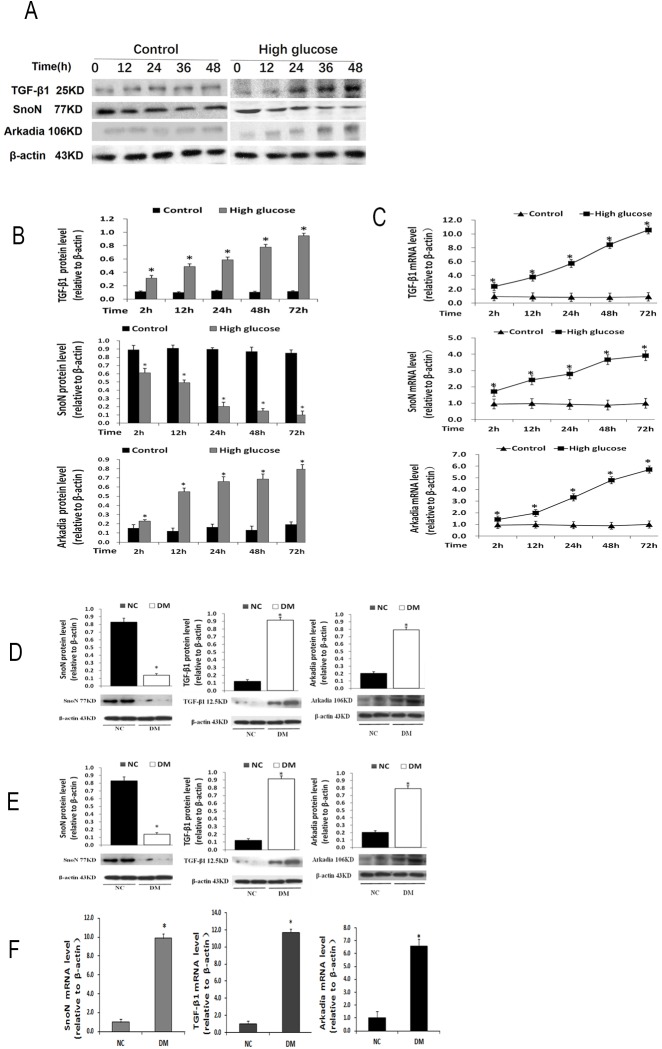
High glucose stress suppresses SnoN while activating TGF-β1 and Arkadia in renal tubular epithelial cells and a rat model of diabetes mellitus. **A, B,** TGF-β1, SnoN and Arkadia protein levels determined by Western blot in NRK52E cells treated with 25 mmol/L glucose for 2, 12, 24, 48 or 72h. *P<0.05 vs respective NRK52E cells treated with 5.5 mmol/L glucose for the same time. **C,** TGF-β1, SnoN and Arkadia mRNA amounts determined by qRT-PCR in NRK52E cells treated with 25 mmol/L glucose for 2, 12, 24, 48 or 72h. *P<0.05, vs. respective NRK52E cells treated with 5.5 mmol/L glucose at 2h. **D,** SnoN, TGF-β1 and Arkadia protein levels determined by Western blot in kidney tissues. *P<0.05, vs. respective normal control group. **E,** SnoN, TGF-β1 and Arkadia mRNA levels determined by qRT-PCR in kidney tissues. *P<0.05, vs. respective normal control group.

We next explored two factors, SnoN and Arkadia, which affect DN progression in the TGF-β1 signaling pathway. NRK52E cells were treated separately in 25 mmol/L glucose (high glucose, HG) and 5.5 mmol/L (normal glucose, NG). In the HG group, SnoN protein levels progressively decreased over time, while TGF- β1 and Arkadia protein amounts were gradually increased, in a time-dependent manner (Fig 3A and 3B). However, the mRNA levels of these three factors (SnoN, TGF- β1, and Arkadia) showed similar increasing trends (Fig 3C).

The above results indicated divergent SnoN expression between the protein and mRNA levels. We then assessed these parameters in a rat diabetic model. The samples collected from DM rat kidney tissues showed significantly decreased SnoN protein levels but increased TGF- β1 and Arkadia protein amounts, in contrast to abundant SnoN protein amounts and sporadic TGF- β1 and Arkadia protein levels in NC samples (Fig 3D). In addition, qRT- PCR analysis of DM rat kidney tissues showed significantly increased mRNA levels of all three factors (Fig 3E). These data were consistent with *in vitro* findings. Taken together, these findings indicated that SnoN is post-transcriptionally regulated.

### SnoN is associated with DN related factors

**Fig 4:**
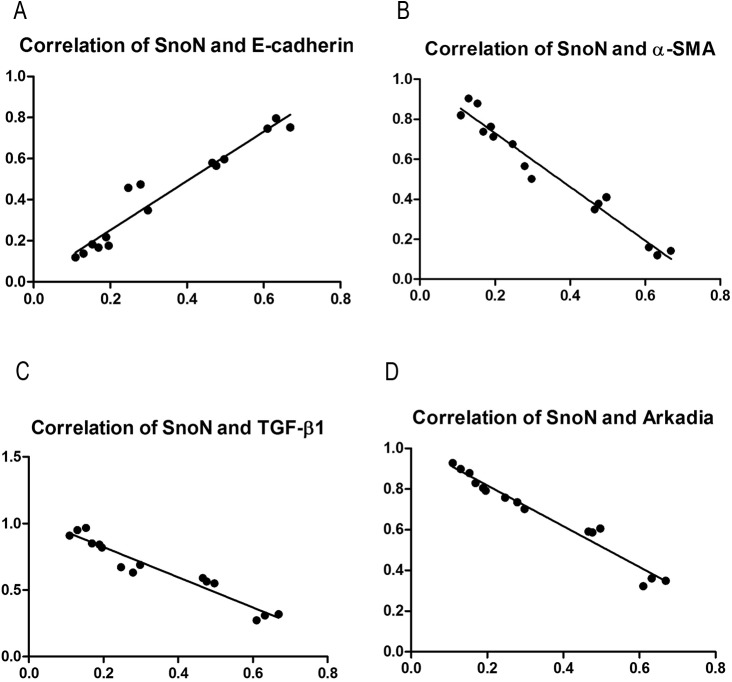
Associations of SnoN with DN related factors. **A,** The expression of SnoN showed significant positive correlation with E-cadherin level (r = 0.733, P<0.01); **B, C, D,** The expression of SnoN showed significant negative correlations with α-SMA, TGF-β1, and Arkadia amounts (r = -0.873, P<0.01; r = -0.769, P<0.01, r = -0.705, P<0.01, respectively)

Protein expression data for SnoN, E-cadherin, α-SMA, TGF-β1, and Arkadia were assessed by SPSS19.0 for potential associations. Interestingly, for samples from NRK52E cells cultured in HG medium, SnoN expression showed a significant positive correlation with E-cadherin level (r = 0.733, P<0.01), and significant negative correlations with α-SMA (r = -0.873, P<0.01), TGF-β1 (r = -0.769, P<0.01), and Arkadia (r = -0.705, P<0.01) (Fig 4A–4D). For samples from diabetic rats, previous studies have found that SnoN was significantly positively correlated with E-cadherin (r = 0.796, P<0.01), and negatively associated with α-SMA (r = -0.882, P<0.01), TGF-β1 (r = -0.671, P<0.01), and Arkadia (r = -0.763, P<0.01) (data not shown).

### SnoN upregulation via Arkadia silencing inhibits EMT in renal tubular cells under high glucose stress

The above findings that SnoN is significantly negatively correlated with TGF-β1 and Arkadia suggested an approach to upregulate SnoN by downregulating Arkadia, considering that Arkadia may represent a better target for therapeutic application compared with TGF-β1. In preliminary experiments, a series of siRNAs targeting Arkadia were assessed, and siRNA-574 was selected for its efficiency (data not shown).

**Fig 5:**
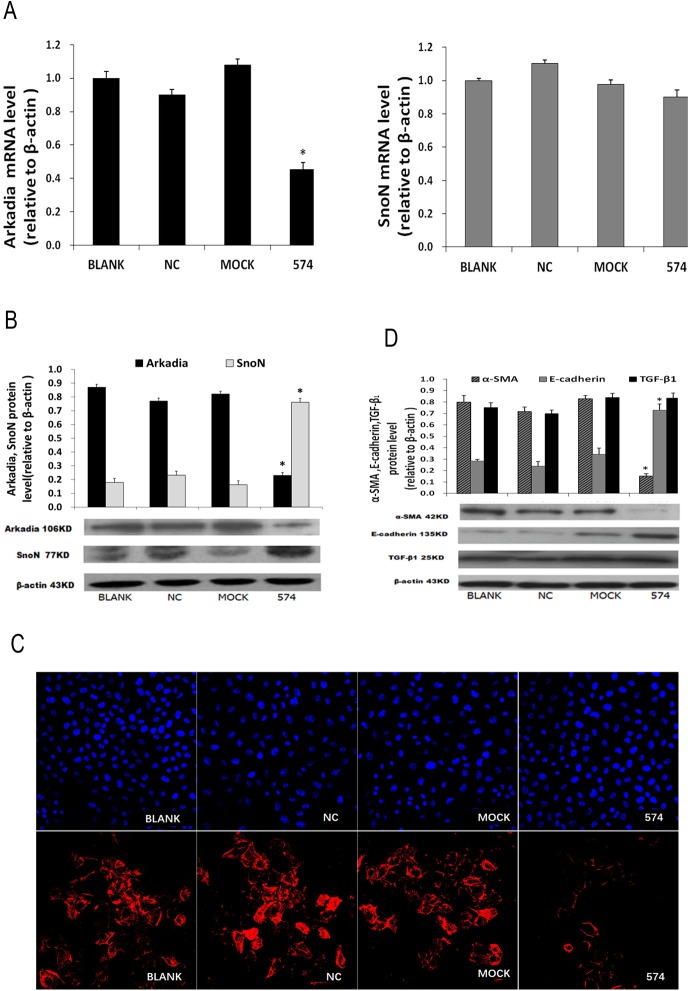
Upregulating SnoN by Arkadia silencing inhibits EMT in renal tubular cells under high glucose stress. **A,** Arkadia and SnoN mRNA levels determined by qRT-PCR. **B,** Arkadia and SnoN protein amounts determined by Western blot. **C,** The expression levels of α-SMA in HK-2 cells assessed by fluorescence microscopy (200×) (red, α-SMA; blue, nucleus). **D,** E-cadherin, α-SMA and TGF-β1 protein levels determined by Western blot. *P<0.05, vs. BLANK.

After transfection with siRNA-574 and culture in HG media for 48h, HK-2 cells were collected for qRT-PCR, western blot, immunofluorescent staining, and ELISA. Compared with the NC and Mock groups, significantly decreased Arkadia mRNA levels and unchanged SnoN amounts were obtained; at the protein level, significantly decreased Arkadia amounts and increased SnoN levels were found (Fig 5A–5C). In addition, the cells transfected with siRNA-574 showed significantly decreased α-SMA and FN levels, and markedly increased E-cadherin amounts. Meanwhile, TGF- β1 protein amounts were not affected after siRNA-574 transfection (Fig 5D; Table 2).

**Table 2:** Protein expression of FN in HK-2 cells as determined by ELISA ($\bar{x}\pm s$, n = 9).

	BLANK	NC	Mock	574
NG(48h)	35.61 ± 3.76	42.77 ± 4.23	37.50 ± 4.98	39.72 ± 5.16
HG(48h)	129.21 ± 7.59*	137.51 ± 7.31*	120.58 ± 6.42*	85.36 ± 4.09*#

Notes

*P<0.05 compared to the NG-BLANK group

^#^P <0.05 compared to the HG-BLANK group.

### Dominant expression of SnoN protects renal tubular cells from EMT under high glucose stress

**Fig 6:**
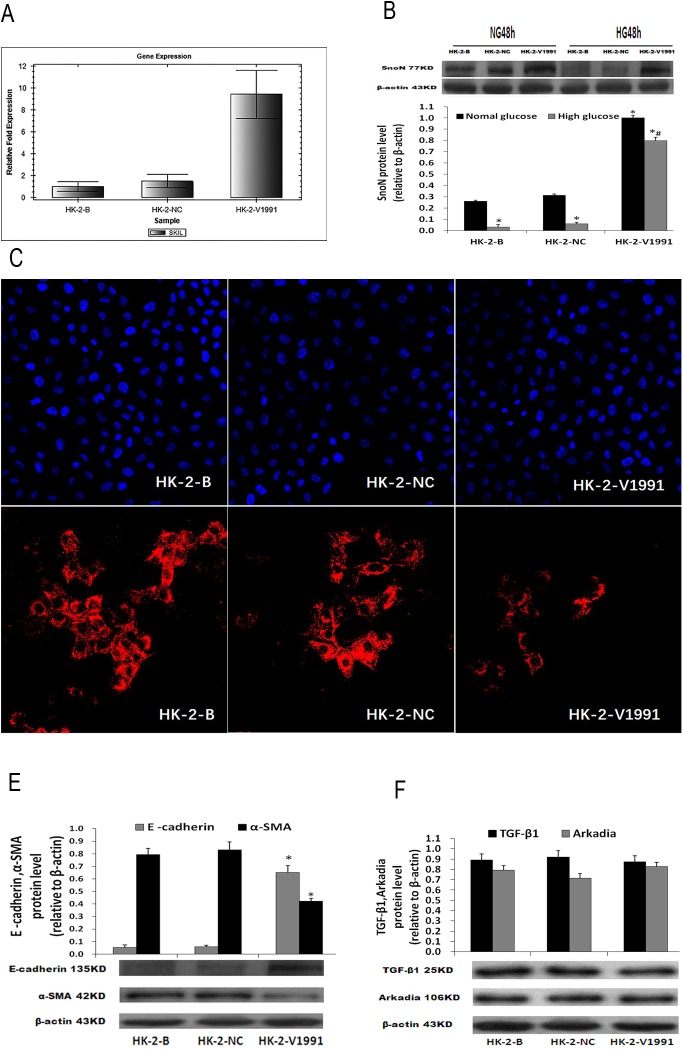
Dominant expression of SnoN protects renal tubular cells from EMT under high glucose stress. **A,** Gene expression levels of hSKIL in HK-2 cells after transfection (72h). **B,** Protein expression of SnoN in HK-2 cells treated with 25 mmol/L glucose or 5.5 mmol/L glucose for 48h. **C,** Expression of α-SMA in HK-2 cells treated with 25 mmol/L glucose for 48h assessed under an inverted fluorescence microscope (200×) (red, α-SMA; blue, nucleus). **D,** Protein expression levels of E-cadherin and α-SMA in HK-2 cells treated with 25 mmol/L glucose for 48h. **E,** Protein expression levels of TGF-β1 and Arkadia in HK-2 cells treated with 25 mmol/L glucose for 48h. *P <0.05 compared to the NG-HK-2-B group; # P <0.05 compared to the HG-HK-2-B group.

Introducing exogenous SnoN may be an alternative approach to SnoN protein overexpression, to antagonize high glucose-triggered EMT. To test this hypothesis, human renal tubular epithelial HK-2 cells were transduced with lentivirus harboring the human SnoN gene coding sequence. By puromycin selection, a cell line stably expressing SnoN driven by the constant EF1α promoter was established, in which robust expression of SnoN in normal culture conditions was first confirmed (Fig 6A). Then, the expression of SnoN was assessed in HG conditions. The results demonstrated dominant expression of the SnoN protein despite negative regulations to overcome (Fig 6B).

As SnoN had dominant expression in the transduced cells, in HG conditions, significantly increased E-cadherin amounts, and markedly decreased α-SMA and FN levels were observed (Fig 6C and 6D; Table 3). This combined expression pattern indicated that dominant SnoN expression inhibited high glucose-induced renal tubular EMT. However, the aberrant expression of SnoN, in this case, did not interfere with TGF-β1 and Arkadia expression (Fig 6E).

**Table 3:** Expression of FN in HK-2 cells as determined by ELISA ($\bar{x}\pm s$, n = 9).

	HK-2-B	HK-2-NC	HK-2-V1991
NG(48h)	49.10±5.37	53.15±6.04	47.28±5.90
HG(48h)	147.25±8.33*	152.76±8.92*	80.31±5.46*#

Notes

*P <0.05 compared to the NG-HK-2-B group

^#^ P <0.05 compared to the HG-HK-2-B group.

## Discussion

Organ fibrosis results from progressive EMT. A set of biomarkers, including E-cadherin (epithelial cell marker), α-SMA (interstitial cell marker), and FN (the major ECM component), are commonly considered to indicate EMT progression, when their expression patterns happen to shift. EMT progression involves multiple factors interacting with TGF-β1/Smad signaling, directly or not. Therefore, tracking EMT markers and understanding the factors involved in TGF-β1/Smad signaling can help evaluate renal fibrosis treatment [17,18]. In this study, we evaluated SnoN due to its role as a negative regulator of TGF-β1.

As shown above, abundant SnoN and E-cadherin amounts, but low TGF-β1, α-SMA and FN levels were detected in normal rat kidney tissues and NRK52E cells cultured in normal glucose media. Meanwhile, significantly decreased SnoN and E-cadherin amounts, alongside starkly increased TGF-β1, α-SMA and FN levels, were determined in DM rat kidney tissues.

Additionally, progressively decreased SnoN and E-cadherin amounts, and gradually increased TGF-β1, α-SMA and FN levels were detected in NRK52E cells cultured in high glucose media. Correlation analysis confirmed that SnoN was positively correlated with E-cadherin but negatively with TGF-β1, α-SMA and FN. These results indicated that the progression of DN-triggered renal fibrosis could be attributed not only to a level increase of the profibrotic TGF-β1 but also a decrease of the antifibrotic SnoN; indeed, decreased SnoN levels may provide substantial contribution to the progression of DN-triggered renal fibrosis.

TGF-β1 mediates SnoN degradation through the ubiquitin proteasome pathway, relying on the formation of the SnoN-Arkadia complex, in which Arkadia acts as a negative regulator of SnoN [19–21]. Previous studies demonstrated that Arkadia is associated with the SnoN protein in free form, as well as in complexes with the Smad2/3 proteins [22–24]. Therefore, we hypothesized that downregulation of Arkadia would, in turn, upregulate SnoN. Arkadia silencing with siRNA provided data supporting the above hypothesis. With the validated siRNA-574, mRNA and protein levels of Arkadia were reduced to 47.68% and 42.59%, respectively (data not shown). Knockdown of Arkadia resulted in significantly increased SnoN amounts, with events characteristic of EMT suppression, including E-cadherin upregulation as well as α-SMA and FN downregulation, appearing simultaneously. This demonstrated the potent suppression of high glucose-induced renal tubular EMT by upregulating SnoN.

The increase of SnoN due to Arkadia knockdown is subject to siRNA efficiency. The efficiency of siRNA-574 in knocking down Arkadia was around 40%. Introducing exogenous SnoN as an alternative approach could be expected to produce higher expression levels. Among the current gene delivery techniques, lentivirus is featured for efficient transduction, stable expression and multi-copy integration. In addition, lentiviral packaging has been normalized for industrial manufacturing and clinical application. In this study, we transduced lentivirus packaged SnoN into human renal tubular cells, and high glucose-induced EMT progression was altered. Quantitative analysis revealed this approach of upregulating SnoN through lentivirus delivery was superior to siRNA knockdown.

In summary, this study demonstrated that either knockdown of the SnoN negative regulator Arkadia or overexpressing exogenous SnoN would increase SnoN expression in renal tubular cells and ameliorate renal fibrosis. In addition, lentiviral transduction showed a better potency than siRNA transfection in this case. Furthermore, SnoN upregulation did not affect the expression of TGF-β1. Overall, these findings provide reliable evidence for developing future treatments of DN targeting SnoN, indicating emerging approaches of engineering ubiquitin-resistant SnoN for treating DN.

## Supporting information

S1 File Animal Experimental Ethical Inspection Form. (PDF)

S2 File Certificate of English Editing. (PDF)

## References

[1] Declèves AE, Sharma K: New pharmacological treatments for improving renal outcomes in diabetes. Nat Rev Nephrol 6: 371–380, 2010 10.1038/nrneph.2010.57 20440278

[2] Herbach N. Pathogenesis of diabetes mellitus and diabetic complications. Studies on diabetic mouse models. Der Pathologe. 2012 Suppl 2:318–24. 23052340 10.1007/s00292-012-1637-1

[3] Kolset SO, Reinholt FP, Jenssen T. Diabetic nephropathy and extracellular matrix. Journal of Histochemistry and Cytochemistry. 2012;60(12):976–986. 10.1369/0022155412465073 23103723 PMC3527883

[4] Sun YM, Su Y, Li J, Wang LF. Recent advances in understanding the biochemical and molecular mechanism of diabetic nephropathy. Biochemical and Biophysical Research Communications. 2013;433(4):359 Bioph 10.1016/j.bbrc.2013.02.120 23541575

[5] Bottinger EP. TGF-beta in renal injury and disease. Semin Nephrol. 2007; 27:309n320. 10.1016/j.semnephrol.2007.02.009 17533008

[6] Lan HY. Diverse roles of TGF-β/Smad in renal fibrosis and inflammation. Int J Biol Sci. 2011; 7:1056–1067. 21927575 10.7150/ijbs.7.1056 PMC3174390

[7] Hills CE, Squires PE. TGF-beta1-induced epithelial-to-mesenchymal transition and therapeutic intervention in diabetic nephropathy. Am J Nephrol. 2010;31(31):68–74. 19887790 10.1159/000256659

[8] Hills CE, Squires PE. The role of TGF-β and epithelial-to mesenchymal transition in diabetic nephropathy. Cytokine Growth Factor Rev. 2011;22(3):131–139. 10.1016/j.cytogfr.2011.06.002 21757394

[9] Liu Y. New insights into epithelial-mesenchymal transition in kidney fibrosis. J. Am. Soc. Nephrol. 2010;21(2):212–22. 10.1681/ASN.2008121226 20019167 PMC4554339

[10] Deheuninck J, Luo K. Ski and SnoN potent negative regulators of TGF-beta signaling. Cell Res. 2009;19(1):47–57. 10.1038/cr.2008.324 19114989 PMC3103856

[11] Luo K. Ski and SnoN negative regulators of TGF-beta signaling. Curr Opin Genet Dev. 2004;14(1):65–70. 10.1016/j.gde.2003.11.003 15108807

[12] Tan R, Zhang J, Tan X, Zhang X, Yang J, Liu Y. Downregulation of SnoN expression in obstructive nephropathy is mediated by an enhanced ubiquitin-dependent degradation. Journal of the American Society of Nephrology. 2006;17(10):2781–2791. 10.1681/ASN.2005101055 16959829

[13] Liu L, Wang Y, Yan R, Li S, Shi M, Xiao Y, et al Oxymatrine Inhibits Renal Tubular EMT Induced by High Glucose via Upregulation of SnoN and Inhibition of TGF-β1/Smad Signaling Pathway. PLoS One. 2016; 11(3): e0151986 10.1371/journal.pone.0151986 27010330 PMC4807015

[14] Mason RM, Wahab NA. Extracellular matrix metabolism in diabetic nephropathy. J Am Soc Nephrol. 2003;14(5):1358–1373. 12707406 10.1097/01.asn.0000065640.77499.d7

[15] Liu R, Wang Y, Xiao Y, Shi M, Zhang G, Guo B. SnoN as a Key Regulator of the High Glucose-Induced Epithelial-Mesenchymal Transition in Cells of the Proximal Tubule. Kidney Blood Press Res. 2012;35(6):517–528. 10.1159/000339172 22813962

[16] Hills CE, Squires PE. TGF-beta1-induced epithelial-to-mesenchymal transition and therapeutic intervention in diabetic nephropathy. The American Journal of Nephrology. 2009;31(1):68–74. 10.1159/000256659 19887790

[17] Bonventre JV. Can we target tubular damage to prevent renal function decline in diabetes? Seminars in Nephrology. 2012;32(5):452–562. 10.1016/j.semnephrol.2012.07.008 23062986 PMC3595316

[18] Cao CX, Yang QW, Lv FL, Cui J, Fu HB, Wang JZ. Reduced cerebral ischemia-reperfusion injury in Toll-like receptor 4 deficient mice. Biochemical and Biophysical Research Communications.2007; 353(2):509–514. 10.1016/j.bbrc.2006.12.057 17188246

[19] Nagano Y, Mavrakis KJ, Lee KL, Fujii T, Koinuma D, Sase H, et al adia induces degradation of SnoN and c-Ski to enhance transforming growth factor-beta signaling.J. Biol. Chem. 2007; 282(28):20492–20501. 10.1074/jbc.M701294200 17510063

[20] Levy L, Howell M, Das D, Harkin S, Episkopou V, Hill CS. Arkadia activates Smad3/Smad4-dependent transcription by triggering signal-induced SnoN degradation. Mol. Cell. Biol. 2007;27(17):6068–6083. 10.1128/MCB.00664-07 17591695 PMC1952153

[21] Le Scolan E, Zhu Q, Wang L, Bandyopadhyay A, Javelaud D, Mauviel A, et al Transforming growth factor-beta suppresses the ability of Ski to inhibit tumor metastasis by inducing its degradation. Cancer Res. 2008;68(9):3277–3285. 10.1158/0008-5472.CAN-07-6793 18451154

[22] Briones-Orta MA, Levy L, Madsen CD, Das D, Erker Y, Sahai E, et al Arkadia regulates tumor metastasis by modulation of the TGF-β pathway. Cancer Research. 2013;73(6):1800–1810. 10.1158/0008-5472.CAN-12-1916 23467611 PMC3672972

[23] Miyazono K, Koinuma D. Arkadia-beyond the TGF-β pathway. Journal of Biochemistry.2011;149(1):1–3. 10.1093/jb/mvq133 21109559

[24] Moustakas A, Heldin CH. Coordination of TGF-β signaling by ubiquitylation. Molecular Cell.2013;51(5):555–556. 10.1016/j.molcel.2013.08.034 24034692

