# Supplementary material for: SnoN upregulation ameliorates renal fibrosis in diabetic nephropathy
Source: PLoS One. 2017 Mar 28;12(3):e0174471. doi: 10.1371/journal.pone.0174471 (PMC5370123; doi:10.1371/journal.pone.0174471)
Supplement: S1 File — (PDF) [file pone.0174471.s001.pdf]

贵州醫科大學
GUIZHOU MEDICAL UNIVERSITY

动物实验伦理审查表

Animal Experimental Ethical Inspection Form of Guizhou Medical University

编号 (No): №1301027

申请人填写的信息 (Related information filled by applicant)

申请单位<br>Name of organization	贵州医科大学病理生理学教研室<br>Department of Pathophysiology, Guizhou Medical University.		
项目经费来源<br>Funding source	国家自然基金项目（编号：81160094）<br>The National Natural Science Foundation of China (Grant No. 81160094)		
申请人<br>Applicant	刘丽荣<br>Lirong Liu	联系电话<br>Telephone	13639077625
实验名称<br>Experiment title	上调 SnoN 减弱糖尿病肾病引起的肾脏纤维化<br>Upregulating SnoN Ameliorates Renal Fibrosis in Diabetic Nephropathy		
申请日期<br>Applicantion date	2013 年 0 1 月 26 日	拟实验时间<br>Experiment date	2013 年 05 月 20 日<br>至 2014 年 01 月 30 日
使用动物情况 动物来源<br>Source of animal	由北京华阜康生物科技股份有限公司<br>HFK Bioscience, Beijing		
使用动物情况 品种品系<br>Species of strain	SD 雄性大鼠<br>Sprague-Dawley rats	等级<br>Grade	清洁级<br>clean grade
使用动物情况 数量<br>Number	♂ 20 只<br>♀ 0 只<br>共 20 只	规格<br>Specifications	180± 20 g
实验设施合格证编号<br>Reg. No. of Experimental Facilities certification	SYXK(黔)2012-0001	质量合格证编号<br>Reg. No. of Quality certification	SCXK(京)2009-0004

实验要点：包括实验目的、实验方法、观测指标、实验结束后处死动物的方法等
Outline of experiments, including aim of experiment , experimental methods, observational index, executing animal method, et al.

本课题拟研究上调 SnoN 是否能够减弱糖尿病肾病引起的肾脏纤维化。SD 大鼠以 55mg/kg 尾静脉注射 STZ 复制 DM 大鼠模型，48 小时后测空腹血糖，血糖≥16.7 mmol/L 且尿糖阳性者视为造模成功；正常对照组大鼠尾静脉注射 STZ 溶媒。20 只 SD 大鼠随机分为 NC 组和 DM 组，每组 10 只；各组大鼠均给予标准饲料喂养，自由饮水，分别饲养至 16 周麻醉处死，收集标本做后续实验分析。

We aim to evaluate the effects of upregulating SnoN on rat renal fibrosis. SD rats wille be received intraperitoneal STZ injections (55 mg/kg). Blood glucose levels be monitored 48 h post injection. Rats with fasting blood glucose levels ≥16.7 mmol/L wille be considered diabetic, and categorized into DM group. Rats in the normal control group will be received sterile citric acid/sodium citrate buffer only. Each group had 10 rats. Animals will be euthanized at week 16, and collect samples for subsequent analysis.

（请翻看背面）

申请者声明<br>Announcement of applicant	我将自觉遵守实验动物福利伦理原则，随时接受实验动物伦理委员会的监督与检查，如违反规定，自愿接受处罚。<br>I will abide by the rules of animal experimental ethics, accept the supervision and inspection of the animal experimental ethics committee, and accept the punishment in case of any infringement.)<br>申请者签名：刘丽菜<br>2013 年 01 月 26 日	
审查依据<br>Inspection contents	1. 该项目是否必须用实验动物进行实验，即能否用计算机模拟、细胞培养等非生命方法替代动物或用低等动物替代高等动物进行实验(Does laboratory animal must be used in the project? Could other methods such as computer simulation. cell culture or using the low-grade animal instead of the high—grade animal?)<br>2. 表中所填申请人资格和所用动物的品种品系、质量等级、规格是否合适，能否通过改良设计方案或用高质量的动物来减少所用动物的数量(Are the qualification of applicant, species or strain, grade and specifications of animals suitable? Could the quantity of animals be reduced by improving the study design or using high quality animals?)<br>3. 能否通过改进实验方法、调整实验观测指标、改良处死动物的方法，来优化实验方案、善待动物(Could the study design and animal treatment be refined by ameliorating experimental method, adjusting observational index. executing animal method?)	
审查结果<br>Results of inspection	课题负责人意见<br>Project director attitude	同意<br>签名：[illegible]
	实验动物伦理委员会委员意见<br>Members attitude of the Animal Care Welfare Committee	同意<br>签名：[illegible]
	实验动物伦理委员会意见<br>Attitude of the Animal Care Welfare Committee	贵州医科大学<br>Guizhou Medical University<br>实验动物伦理委员会<br>the Animal Care Welfare Committee<br>主任委员签名：[illegible]<br>盖章<br>年 月 日
备注:<br>Remark		

说明：

1. 申请表（包括必要的审查资料）纸质版三份递交到贵州医科大学实验动物中心，电子版发送到gmcllsc@163.com，编号由实验动物中心分配并填写。
2. 课题负责人、执行人及合作单位负责人均需在声明人签字栏签字。
3. 需在外单位完成课题的，请同时填写校外实验动物设施使用证明。
4. 表格所有填写内容请用签字笔填写或电脑打印(签名处除外)。
5. 需随本表递交相关审查资料如实验方案、课题标书（复印件）等。要求写明项目的意义、必要性、项目中有关实验动物的用途、饲养管理或实验处置方法、预期出现的对动物的伤害、处死动物的方法、项目进行中涉及动物福利的详细描述。
