# Supplementary material for: SnoN upregulation ameliorates renal fibrosis in diabetic nephropathy
Source: PLoS One. 2017 Mar 28;12(3):e0174471. doi: 10.1371/journal.pone.0174471 (PMC5370123; doi:10.1371/journal.pone.0174471)
Supplement: S2 File — (PDF) [file pone.0174471.s002.pdf]

MedSci

Editing by professional editors

# CERTIFICATE OF ENGLISH EDITING

Date: 06, Dec, 2016

This document certifies that the manuscript titled " SnoN Upregulation Ameliorates Renal Fibrosis in Diabetic Nephropathy " was edited for proper English language, grammar, punctuation, spelling, and overall style by one or more of the highly qualified native English speaking editors at MedSci. Neither the research content nor the authors' intentions were altered in any way during the editing process.

Documents receiving this certification should be English-ready for publication - however, the author has the ability to accept or reject our suggestions and changes. To verify the final MedSci edited version, please visit our verification page. If you have any questions or concerns over this edited document, please contact MedSci at editing@medsci.cn

**Manuscript title:** SnoN Upregulation Ameliorates Renal Fibrosis in Diabetic Nephropathy

**First Author:** Lirong Liu

**Code:** 0612-6CBC-D79A-9A85-4153

This certificate may be verified at www.medsciediting.com/certificate/

MedSci affiliated to BIOON Group, offers the services in paper evaluation, professional translation, language editing and publication support.

MedSci comprises a network of more than 300 editors with a biomedical background. All our editors must meet strict selection criteria. We require that each editor has completed or is attending a PhD graduate program at one of the top universities in the world. He or she must have proven written communication skills and undergo a comprehensive training period under the instruction of a managing editor. All editors are native English speakers.

We provide high quality services at the comparable price in the industry. For more information, please visit www.medsciediting.com or contact us by email at editing@medsci.cn
